# Polyfunctional CD4^+^ T Cell Responses to Immunodominant Epitopes Correlate with Disease Activity of Virulent *Salmonella*

**DOI:** 10.1371/journal.pone.0043481

**Published:** 2012-08-17

**Authors:** Matt Maybeno, Anke Redeker, Suzanne P. M. Welten, Bjoern Peters, Scott M. Loughhead, Stephen P. Schoenberger, Alessandro Sette, Ramon Arens

**Affiliations:** 1 Division of Vaccine Discovery, La Jolla Institute for Allergy and Immunology, La Jolla, California, United States of America; 2 Laboratory of Cellular Immunology, La Jolla Institute for Allergy and Immunology, La Jolla, California, United States of America; 3 Department of Immunohematology and Blood Transfusion, Leiden University Medical Center, Leiden, The Netherlands; MRC National Institute for Medical Research, United Kingdom

## Abstract

*Salmonella enterica* serovars are intracellular bacteria capable of causing typhoid fever and gastroenteritis of significant morbidity and mortality worldwide. Current prophylactic and therapeutic treatment is hampered by the emergence of multidrug-resistant (MDR) strains of *Salmonella*, and vaccines provide only temporal and partial protection in vaccinees. To develop more effective *Salmonella* vaccines, it is important to understand the development of protective adaptive immunity to virulent *Salmonella*. Here we report the identification of novel CD4^+^ T cell peptide epitopes, which are conserved among *Salmonella* serovars. Immunization of *Salmonella*-infected mice with these peptide epitopes reduces the burden of *Salmonella* disease. Furthermore, we show that distinct polyfunctional (interferon-γ^+^, tumor necrosis factor^+^, and interleukin-2^+^) *Salmonella*-specific CD4^+^ T cell responses develop with respect to magnitude and kinetics. Moreover, we found that CD4^+^ T cell responses against immunodominant epitopes are predictive for active *Salmonella* disease. Collectively, these data could contribute to improved diagnosis of *Salmonella*-related diseases and rational design of *Salmonella* vaccines.

## Introduction

*Salmonella enterica* serovars are intracellular bacteria capable of causing localized and systemic disease of significant morbidity and mortality [1], [2], [3]. Natural acquisition of *Salmonella* via contaminated water or food introduces the bacteria to the gastrointestinal tract, where *Salmonella* colonizes underlying mucosal tissue and disseminates to spleen and liver [4], [5]. *Salmonella enterica* serovar Typhi causes typhoid fever in humans with an estimated 21 million cases annually, resulting in more than 200,000 deaths per year in endemic areas [6], [7]. The closely related *Salmonella enterica* serovar Typhimurium and Enteriditis cause gastroenteritis, a foodborne disease which constitutes a major public health burden and represents a significant cost to society in many countries [2], [8]. Thus, infections with *Salmonella* (salmonellosis) are not only a health concern in developing nations but are also an important cause of gastrointestinal infections in developed nations where contaminated food products are rapidly and widely distributed.

Despite the use of antibiotics in recent years, salmonellosis remains a major public health problem, both in terms of incidence and severity of cases. Many typhoid patients in developing countries fail to recover due to lack of treatment or a substantial delay in antibiotic administration [1], [3]. In addition, multidrug-resistant (MDR) strains of *Salmonella* often emerge [9]. Although subunit vaccines comprising antigens conserved across different *Salmonella* serovars have the potential to be a safe and cost-effective prophylactic measure in combating *Salmonella*-related diseases, the current available vaccines provide only 50–60% protection in vaccinated individuals [3], [7]. Moreover, typhoid vaccines lose effectiveness after several years. Importantly, the immune mechanisms mediating protection by such vaccination are not well understood, and which antigens are important for the induction of immunity to *Salmonella* is largely unknown.

In experimental models of *Salmonella* infection it was shown that mice develop a typhoid-like disease following *S*. Typhimurium infection [10]. Mice lacking MHC class II-restricted CD4^+^ T cells and B cells due to knockout mutations or antibody depletion are highly susceptible to both attenuated and virulent *Salmonella* [11], [12], [13], [14], indicating important functions of these cells for immunity. In contrast, mice lacking class I-restricted CD8^+^ T cells or γδ T cells can resolve infection with attenuated strains [12]. The mechanisms by which *Salmonella*-specific CD4^+^ T cells contribute to protective immunity are incompletely understood [15] and attempts to transfer immunity with *Salmonella*-specific T cell lines have failed to identify the specificity of the response [16], [17]. The repertoire of *Salmonella* epitopes recognized by CD4^+^ T cells during infection is largely unknown with the exception of epitopes in flagellin (FliC) [18], [19], [20] and the type-III-secretion system [21].

A hurdle to the development of effective vaccines against (chronic) infectious diseases is the identification of antigens capable of eliciting polyfunctional T cell responses that provide immune correlates for disease activity and/or vaccine-mediated protection. In this study, we have identified novel CD4^+^ T cell epitopes that are conserved among *Salmonella* serovars. Furthermore, we show that diverse kinetics and polyfunctional profiles of *Salmonella*-specific CD4^+^ T cell responses in mice exist, and that the CD4^+^ T cell responses to immunodominant epitopes are predictive for disease activity. These data could lead to improved diagnosis of *Salmonella*-related diseases and enhance rational design of vaccines against *Salmonella* serovars.

## Results

### CD4^+^ T cell responses control virulent *Salmonella* infection

**Figure 1:**
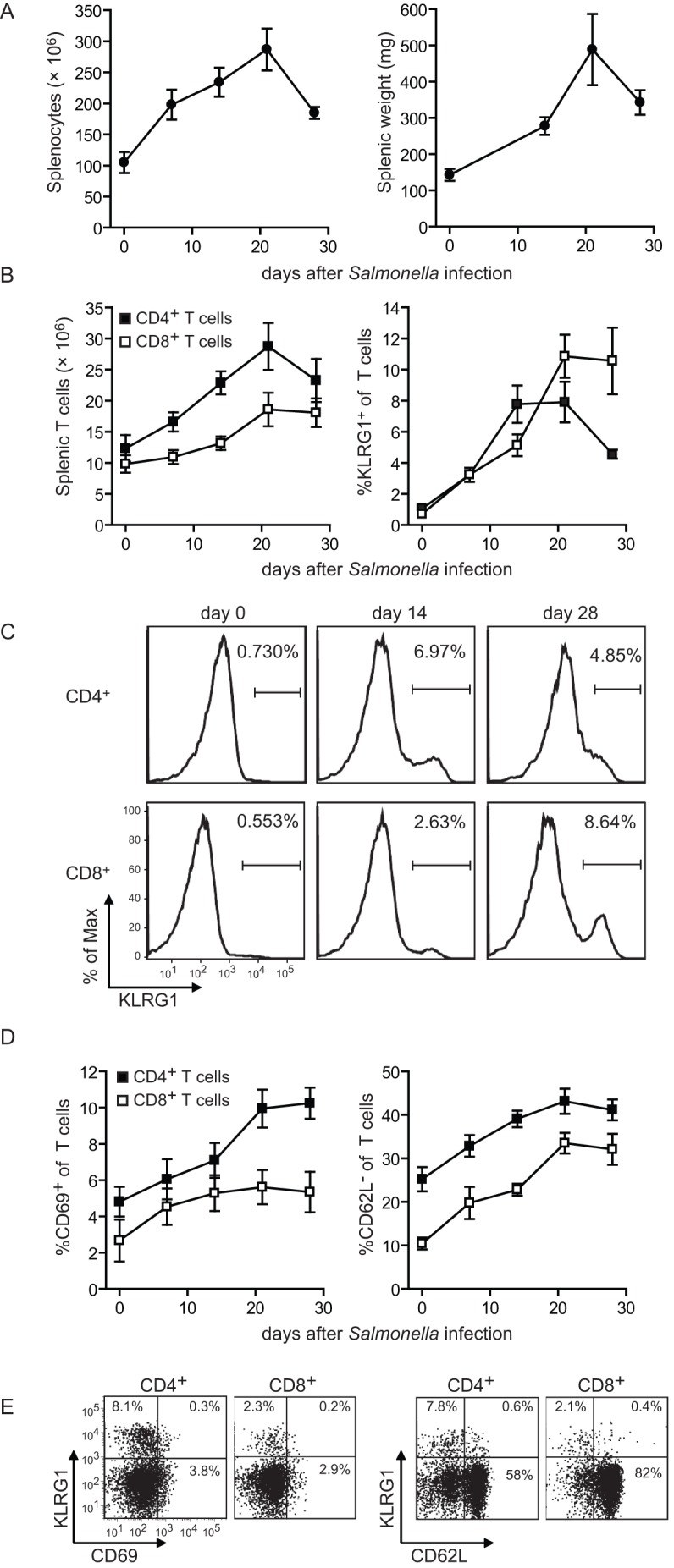
*Salmonella* infection induces splenomegaly and activation of T cells. C57BL/6 mice were infected orally with 1×10^7^ virulent *Salmonella* Typhimurium (strain LT2). At day 7, 14, 21 and 28 post-infection, (A) the total number of splenocytes and the splenic weight were determined. (B) Graphs show the total number of splenic CD4^+^ (Foxp3^−^) and CD8^+^ T cells and the percentage of KLRG1 within these populations over time. Data are shown as mean ± SEM of 5–7 mice per timepoint. (C) Representative histogram plots show the KLRG1 expression on splenic CD4^+^ (Foxp3^−^) and CD8^+^ T cells at the indicated timepoints post-infection. Numbers indicate the percentage of KLRG1^+^ cells within the CD4^+^ (Foxp3^−^) or CD8^+^ T cell population. (D) Graphs show the percentage of CD69^+^ and CD62L^−^ within the CD4^+^ (Foxp3^−^) and CD8^+^ T cell populations over time. Each data point shows mean ± SEM for 5 mice per group. (E) Representative dot plots show the KLRG1 *versus* CD69 or the KLRG1 *versus* CD62L expression on splenic CD4^+^ (Foxp3^−^) and CD8^+^ T cells at day 14 post-infection. The percentage of positive cells within each quadrant is indicated. Experiments were performed twice with similar results.

We initially set up a natural course of virulent *Salmonella* infection in the susceptible mouse strain C57BL/6 with the *Salmonella enterica* serovar Typhimurium (*S*. Typhimurium) strain LT2. After oral infection, which mimics the natural route of infection, with 1×10^7^ *S*. Typhimurium the absolute number of splenocytes and splenic weight in *Salmonella* infected mice progressively increased until week 3 post-infection (Figure 1A). At week 4 post-infection, the total splenocyte number and splenic weight declined (Figure 1A). The majority of the mice became moribund at later timepoints (5–6 weeks post-infection), and had to be euthanized because of ethical reasons. In parallel, we evaluated the magnitude and activation status of CD4^+^ and CD8^+^ T cell populations during the course of infection. The absolute number of CD4^+^ and CD8^+^ T cells followed similar kinetics as the total number of splenocytes (Figure 1B). The expression of killer cell lectin-like receptor G1 (KLRG1), which identifies antigen-experienced T cells [22], [23], was progressively increased on the cell surface of both CD4^+^ (Foxp3^−^) and CD8^+^ T cells and showed similar kinetics as the absolute lymphocyte counts (Figure 1B and 1C). Other indicators of T cell activation such as the up-regulation of the early activation marker CD69 and the down-regulation of the lymph node homing molecule CD62L were also observed during the course of infection (Figure 1D and 1E). Thus virulent *Salmonella* infection expands T cell populations that display evidence of activation and likely represents development of *Salmonella*-specific T cell responses.

**Figure 2:**
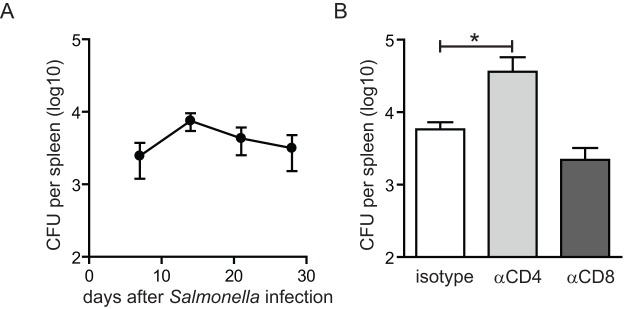
CD4^+^ T cells reduce the burden of virulent *Salmonella* infection. C57BL/6 mice were infected orally with 1×10^7^ virulent *Salmonella* Typhimurium. (A) At day 7, 14, 21 and 28 post-infection, the *Salmonella* CFU in the spleen were determined. Data show the mean ± SEM of 5–7 mice per timepoint. (B) At the onset of *Salmonella* infection, CD4^+^ or CD8^+^ T cell populations were depleted with anti-CD4 (clone GK1.5) or anti-CD8 (clone 2.43) monoclonal antibodies and at day 14 post-infection recoverable *Salmonella* CFU were determined. Graph shows mean + SEM for 5–6 mice per group. Statistical significance between groups is indicated (Mann-Whitney test, *p<0.05). Experiments were performed twice with similar results.

The bacterial burden increased until week 2 post-infection after which the number of live bacteria remained relatively high (Figure 2A). To examine the importance of activated CD4^+^ and CD8^+^ T cell populations for controlling virulent *Salmonella* infection, we selectively depleted these subsets by using depleting monoclonal antibodies. The depletion of CD4^+^ T cells after the onset of infection resulted in significantly increased numbers of *Salmonella* bacteria, whereas CD8^+^ T cell depletion did not (Figure 2B). Thus, although both CD4^+^ and CD8^+^ T cell subsets are activated, CD4^+^ T cells play a particular important role in the control of virulent *Salmonella* infection. Together these data establish the tempo of persistent infection, the accompanied activation status of the T cells, and the importance of CD4^+^ T cells in our experimental virulent *Salmonella* infection model and reflects the findings of other *Salmonella* infection models [11], [12], [13], [24], [25], [26], [27].

### Identification of peptide epitopes to track *Salmonella*-specific CD4^+^ T cell responses

Given the importance of CD4^+^ T cells during *Salmonella* infection, we predicted genome-wide the MHC class II (H2 I-A^b^) binding peptides encoded by *S*. Typhimurium strain LT2 [28] and *S*. Typhi strain CT18 [29] using optimized computer-based algorithms [30], [31]. Since the amino acid homology of the *S*. Typhimurium and Typhi is >95% analogous, we selected only conserved epitopes. For the screening of the *Salmonella* epitopes, we tested by interferon (IFN)-γ ELISPOT the top 1443 predicted epitope candidates pooled in 180 mixtures of 8 peptides each for their ability to restimulate splenic CD4^+^ T cells of C57BL/6 mice orally infected 18 days earlier with virulent *S*. Typhimurium. To minimize background levels of ELISPOT assays, we loaded *in vitro* cultured dendritic cells (DCs) from naive mice with the peptides and used these peptide-loaded cells to restimulate the splenic CD4^+^ T cells that were purified by magnetic beads. In this screen, we identified three positive pools. Analysis of the individual peptide epitopes of these pools yielded three peptides with positive responses. The identified CD4^+^ T cell epitopes were all novel and were located in different gene products of *Salmonella* namely in the alkyl hydroperoxide reductase subunit C (AhpC), the ethanolamine ammonia-lyase small subunit (EutC) and in STM1540, a putative hydrolase related to choloylglycine hydrolase (Table 1). Previous work showed that expression of AhpC is induced by the oxidative burst of macrophages and that this *Salmonella* enzyme plays a role in the stimulation of T cells [32], [33]. A study using mass spectometry-based proteomics confirmed the obvious presence of AhpC in *Salmonella* and additionally detected expression of EutC [34]. A comprehensive study that performed proteomics and transcriptional analyses showed that expression of AhpC was altered post-transcriptionally through the RNA-binding protein SmpB while post-transcriptional regulation of STM1540 was via the RNA-binding protein Hfq [35]. Blast searches of the amino acid sequences using The National Center for Biotechnology Information (NCBI) web pages and databases revealed that the three newly identified CD4^+^ T cell epitope sequences are not only conserved in *S*. Typhi and Typhimurium but are also conserved among many other *Salmonella* serovars. The AhpC_154–168_ epitope is fully conserved in other microbes as well such as *Pseudomonas*, *Burkholderia*, *Citrobacter* and *Polaromonas*. Also the EutC_243–257_ epitope sequence is 100% conserved in other micro-organisms including *Klebsiella*, *Shigella*, *Citrobacter*, *Escherichia coli* and *Aspergillus* but the amino acid homology of the STM1540_262–276_ epitope is 80% or lower with other bacteria (e.g. *Geobacter* and *Xanthomonas*).

**Table 1:** CD4^+^ T cell epitopes to virulent *Salmonella*.

Protein	Epitope^a^	Sequence	Protein function	ELISPOT^b^
AhpC	154–168	AAQYVAAHPGEVCPA	alkyl hydroperoxide reductase subunit C	5.1
EutC	243–257	CYAVYSPRVATTVEA	ethanolamine ammonia-lyase small subunit	2.4
STM1540	262–276	GVYYTTYAPQATSAH	putative hydrolase	17.4

a Indicated is the amino-acid position in *Salmonella* Typhimurium strain LT2 [28].

b Mice were infected orally with 1×10^7^ *Salmonella* Typhimurium (strain LT2). After 18 days, CD4^+^ T cells were tested in ELISPOT assays for IFN-γ production. Data is presented as Stimulation Index (SI), calculated as the number of spot forming cells after stimulation with peptide-loaded dendritic cells divided by spot forming cells in background.

**Figure 3:**
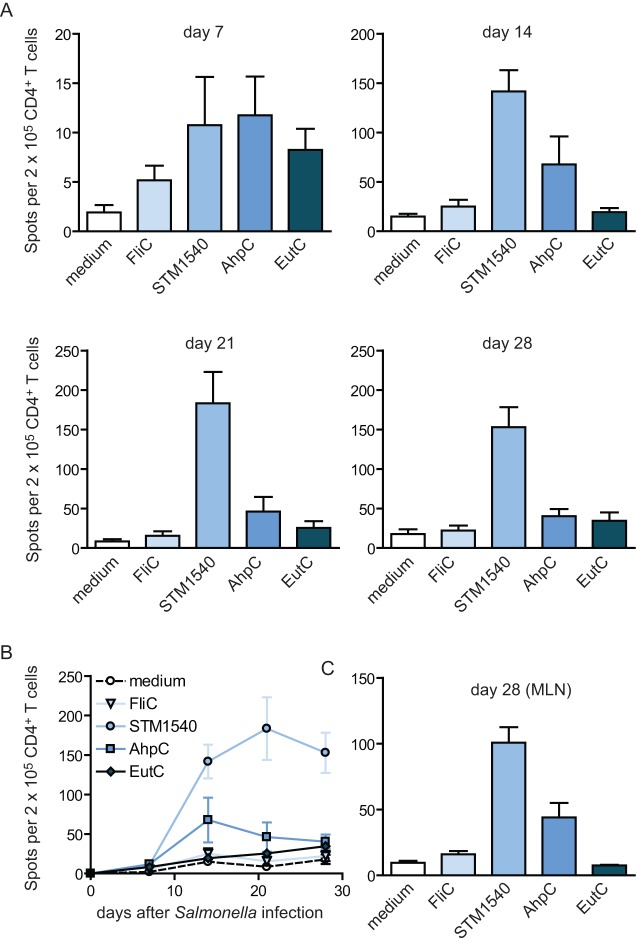
Tracking of CD4^+^ T cell responses during virulent *Salmonella* infection. C57BL/6 mice were infected orally with virulent *Salmonella* Typhimurium and at day 7, 14, 21 and 28 post-infection CD4^+^ T cells were isolated from the spleen and restimulated with dendritic cells that were either loaded with different *Salmonella* peptide epitopes (AhpC_154–168_, EutC_243–257_, STM1540_262–276_ and FliC_429–443_) or not loaded with peptide (medium). (A) Graphs show the number of IFN-γ^+^ spots per 2×10^5^ CD4^+^ T cells, as measured by ELISPOT assays, for each epitope at the indicated timepoints post-infection. (B) Graph shows similar data as in (A) depicted as the IFN-γ^+^ spot frequency in time. (C) Graph shows the number of IFN-γ^+^ spots per 2×10^5^ CD4^+^ T cells that were purified from mesenteric lymph nodes (MLN) at day 28 post-infection. All graphs show mean and SEM of 5–6 mice per group. Experiments were performed twice with similar results.

Tracking the *Salmonella*-specific CD4^+^ T cell response over time revealed that the most immunodominant response throughout infection was against the STM1540_262–276_ epitope, followed by AhpC_154–168_ and EutC_243–257_ respectively (Figure 3A and 3B). The response against the previous identified FliC_429–443_ epitope [19] epitope was roughly similar in magnitude as the EutC_243–257_ epitope. Besides displaying variation in magnitude, the kinetics of *Salmonella*-specific CD4^+^ T cell responses were also diverse: whereas STM1540_262–276_-specific CD4^+^ T cell responses increase until day 21, the responses against the AhpC_154–168_ and FliC_429–443_ epitopes contracted after day 14 post-infection. Moreover, the percentage of EutC_243–257_-specific CD4^+^ T cells gradually increased until day 28 post-infection (Figure 3B). The STM1540_262–276_ and AhpC_154–168_-specific CD4^+^ T cell responses were also clearly detectable in the mesenteric lymph nodes of *Salmonella*-infected mice at day 14, 21 and 28 post-infection (Figure 3C and data not shown). Together, these data show that CD4^+^ T cell epitopes to virulent *Salmonella* can be identified and that *Salmonella*-specific CD4^+^ T cell responses are diverse in magnitude and kinetics.

### Peptide vaccination reduces the burden of virulent *Salmonella infection*

**Figure 4:**
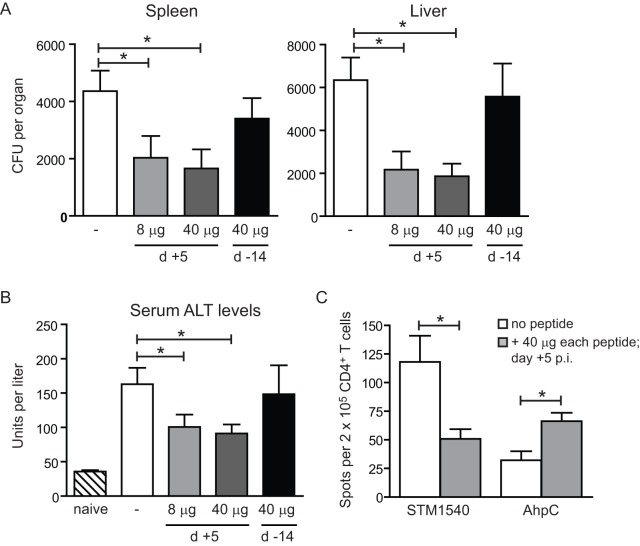
Vaccination with CD4^+^ T cell epitopes during ongoing infection reduces the burden of *Salmonella* disease. C57BL/6 mice were infected orally with 1×10^7^ virulent *Salmonella* Typhimurium. Groups of mice (n = 7–8) were vaccinated with a mixture of the peptides AhpC_154–168_, EutC_243–257_, and STM1540_262–276_ (8 µg (∼5 nmol) or 40 µg (∼25 nmol) of each peptide) at day 5 post-infection (therapeutic setting) or 14 days before *Salmonella* infection (prophylactic setting). Control mice received PBS without peptides. Mice were sacrificed at day 14 post-infection and (A) the *Salmonella* CFU were determined in spleen and liver, and (B) the ALT levels were determined in the serum. (C) CD4^+^ T cells purified from the spleens of the non-vaccinated mice and the mice vaccinated at day 5 post-infection (p.i.) with 40 µg of each of the peptides (AhpC_154–168_, EutC_243–257_, and STM1540_262–276_) were restimulated with dendritic cells that were loaded with the indicated *Salmonella* peptide epitopes. Graph shows the IFN-γ^+^ spots per 2×10^5^ CD4^+^ T cells measured by ELISPOT assays. Statistical significance between the peptide vaccinated and non-vaccinated groups is indicated (Mann Whitney test, *p<0.05). Naive mice (n = 8) were used to determine the normal serum ALT levels. Bar graphs show mean and SEM. Experiments were performed twice with similar results.

To assess whether the newly identified peptide epitopes could elicit *Salmonella*-specific CD4^+^ T cell responses that contribute to immune control, we vaccinated wild-type mice at day 5 post *Salmonella* infection with a mixture of the peptides AhpC_154–168_, EutC_243–257_ and STM1540_262–276_ (8 µg (∼5 nmol) or 40 µg (∼25 nmol) of each peptide). Quantification of the bacterial colonization in the spleens and livers at day 14 post-infection revealed a significant decrease of the bacterial burden in the peptide vaccinated groups as compared to non-vaccinated mice (Figure 4A). The positive impact of the peptide vaccine on *Salmonella* disease was also reflected in the decreased serum level of alanine aminotransferase (ALT) (Figure 4B), which is known to be indicative for liver damage [36] and previously reported to be elevated in human salmonellosis [37], [38]. Analysis of the CD4^+^ T cell responses by ELISPOT revealed that the peptide vaccination resulted in a two-fold increase of the AhpC-specific CD4^+^ T cell response but the STM1540-specific CD4^+^ T cell response was decreased as compared to unvaccinated mice (Figure 4C). Vaccination with the same mix of peptides in a prophylactic setting (i.e. 14 days before *Salmonella* challenge) however, did not result in reduction of bacterial load or ALT levels (Figure 4 and 4B). Together these data demonstrate that boosting of CD4^+^ T cell responses during infection with peptide-based vaccines improves the clearance and burden of virulent *Salmonella* bacteria.

### Virulent *Salmonella* infection elicits polyfunctional CD4^+^ T cell responses

The identification of differential cytokine expressing CD4^+^ T cells could lead to novel predictors of disease activity of virulent *Salmonella* infection as has recently been found for other pathogens such as HIV and *Mycobacterium tuberculosis* [39], [40], [41]. Furthermore, it has been shown that the quality of a CD4^+^ T cell cytokine response is able to define correlates of protection against pathogens [42], [43]. To functionally characterize the CD4^+^ T cell response to virulent *Salmonell*a, we determined by intracellular cytokine staining and polychromatic flow cytometry [44] whether *Salmonella*-specific CD4^+^ T cells can simultaneously produce IFN-γ, tumor necrosis factor (TNF), interleukin (IL)-2, IL-10 and/or IL-17.

**Figure 5:**
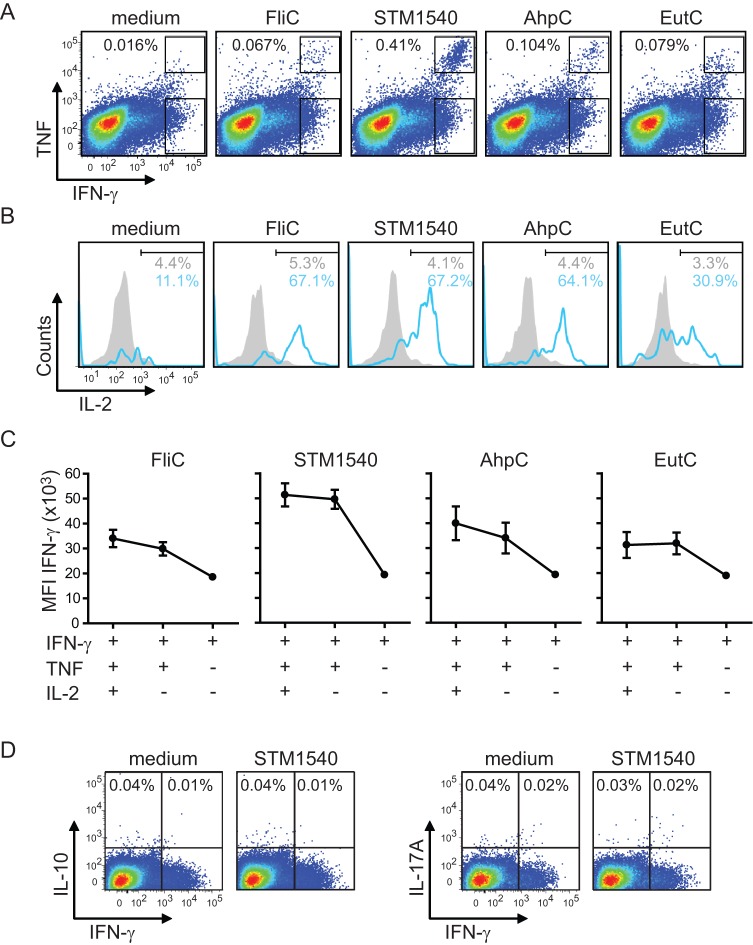
Development of polyfunctional CD4^+^ T cell responses during *Salmonella* infection. C57BL/6 mice were infected orally with 1×10^7^ virulent *Salmonella* Typhimurium and at day 14 post-infection the cytokine profiles of *Salmonella*-specific CD4^+^ T cells were determined by polychromatic flow cytometry. (A) Shown are representative analyses of the IFN-γ and TNF cytokine profiles gated on CD3^+^CD4^+^ T cells, which were stimulated with dendritic cells that were either loaded with peptides (i.e. FliC_429–443_, STM1540_262–276_, AhpC_154–168_ or EutC_243–257_) or not loaded with peptide (medium). The percentage of IFN-γ^+^TNF^+^ within the CD3^+^CD4^+^ T cell population is indicated. (B) Histogram plots show the percentage of IL-2 positive cells within the double IFN-γ^+^TNF^+^ (blue line) and single IFN-γ^+^ (filled grey histograms) population. (C) Shown is the IFN-γ mean fluorescence intensity (MFI) ± SEM of epitope-specific triple IFN-γ^+^TNF^+^IL-2^+^, double IFN-γ^+^TNF^+^ and single IFN-γ^+^ CD3^+^CD4^+^ T cells. (D) Shown are representative analyses of the intracellular IL-10, IL-17A and IFN-γ expression of CD3^+^CD4^+^ T cells, which were stimulated with dendritic cells that were loaded with STM1540_262–276_ peptide or not loaded with peptide (medium). The numbers indicate the percentage of cells that are positive for the indicated cytokines in each quadrant. Four independent experiments were performed with four mice per experiment.

The analysis of intracellular cytokine production by CD4^+^ T cells in which total splenocyte cultures were restimulated with peptide revealed that the background (peptide unstimulated) IFN-γ and TNF staining was very high (data not shown), which is likely attributed to the persistent presence of virulent *Salmonella* bacteria in antigen presenting cells. By using the same stimulation procedure as used in the ELISPOT assays to stimulate the *Salmonella*-specific CD4^+^ T cells (i.e. stimulation of purified CD4^+^ T cells by peptide-loaded DCs) the IFN-γ and TNF background staining decreased considerably. A DC-T cell ratio of 1∶5 and 1∶10 was optimal, but ratios of 1∶20 and 1∶40 also elicited positive responses (Figure S1). Importantly, at two, three and four weeks post-infection a population of high IFN-γ producers was found exclusively in the peptide-stimulated samples, which were more easily visible by costaining for TNF (Figure 5A and data not shown). This indicates that the IFN-γ^+^TNF^+^ CD4^+^ T cells most likely identify the epitope-specific population, and confirms the findings of Srinivasan et al [45]. Further examination of the polyfunctionality of the *Salmonella*-specific CD4^+^ T cells revealed that most of the IFN-γ^+^TNF^+^ cells co-produced IL-2 (Figure 5B), demonstrating a predominant development into polyfunctional T cells with a T-helper type 1 (T_H_1) signature [46]. Consistent with the immunodominance hierarchy, the mean fluorescence intensity (MFI) of IFN-γ within the triple IFN-γ^+^TNF^+^IL-2^+^ and double IFN-γ^+^TNF^+^ CD4^+^ T cells was the highest for the reactivity of these cells against the STM1540_262–276_ epitope followed by AhpC_154–168_, EutC_243–257_ and FliC_429–443_ (Figure 5C). The IFN-γ MFI of the single IFN-γ^+^ CD4^+^ T cells was consistently lower than the triple IFN-γ^+^TNF^+^IL-2^+^ and double IFN-γ^+^TNF^+^ populations. Intracellular expression of IL-10 and IL-17A was neither found in the double producers nor found in the total CD4^+^ T cell population (Figure 5D). Thus, polyfunctional CD4^+^ T cells that develop during *Salmonella* infection produce simultaneously IFN-γ, TNF and IL-2 and the differential amounts of IFN-γ that is being produced by these cells correlates with the immunodominance hierarchy.

### Polyfunctional CD4^+^ T cell responses correlate to disease activity

**Figure 6:**
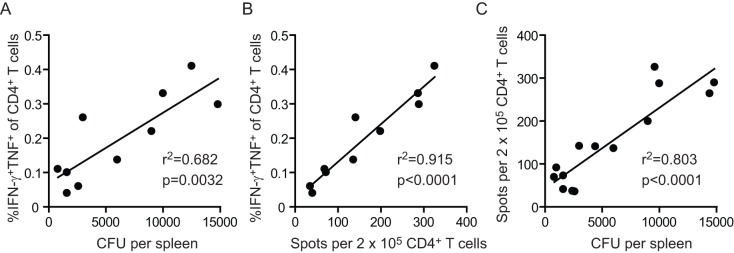
Polyfunctional CD4^+^ T cell responses correlate with disease activity of virulent *Salmonella*. C57BL/6 mice were infected orally with 1×10^7^ virulent *Salmonella* Typhimurium and at day 14 and 21 post-infection the bacterial burden and *Salmonella*-specific CD4^+^ T cell responses were analyzed. (A) Correlation between the frequency of STM1540_262–276_-specific IFN-γ^+^TNF^+^ CD3^+^CD4^+^ T cells and the recoverable *Salmonella* CFU in the spleen. (B) Correlation between the number of IFN-γ spots (as determined by ELISPOT) and the frequency of STM1540_262–276_-specific IFN-γ^+^TNF^+^ CD3^+^CD4^+^ T cells. (C) Correlation between the STM1540_262–276_-specific IFN-γ ELISPOTS and recoverable *Salmonella* CFU in the spleen. Data are cumulative from 12–15 *Salmonella*-infected mice analyzed at day 14 and 21 post-infection. Two independent experiments were performed at each timepoint post-infection with 3–5 mice per experiment. Correlation coefficient and significance value is indicated. Each data point represents an individual mouse.

To determine statistical correlations between the *Salmonella*-specific CD4^+^ T cell responses and the disease activity of *Salmonella*, we plotted the CD4^+^ T cell frequency against the recoverable *Salmonella* CFU in the spleen. There was a significant correlation between the frequency of STM1540_262–276_-specific IFN-γ^+^TNF^+^ CD4^+^ T cells and the splenic bacterial load (Figure 6A). Also the CD4^+^ T cell response against AhpC_154–168_ but not the EutC_243–257_ epitope correlated with the bacterial colonization (data not shown). Plotting of the STM1540_262–276_-specific IFN-γ^+^TNF^+^ CD4^+^ T cell frequency against the number of IFN-γ^+^ spots as determined by ELISPOT showed a strong correlation between these two assays for enumerating antigen-specific T cells (Figure 6B). Accordingly, the *Salmonella*-specific CD4^+^ T cells responses as determined by IFN-γ ELISPOT correlated to the disease activity of *Salmonella* (Figure 6C). In conclusion, these data provide evidence that immunodominant CD4^+^ T cell responses that develop during virulent *Salmonella* infection are predictive for the disease activity.

## Discussion

Our results indicate that polyfunctional CD4^+^ T cell responses, which develop during *Salmonella* infection, can predict important elements of disease activity. Although both CD4^+^ and CD8^+^ T cell populations were activated, the depletion of CD4^+^ but not CD8^+^ T cells affected bacterial clearance. The importance of CD4^+^ T cell activation was further demonstrated by the efficacy of therapeutic vaccination with the CD4^+^ T cell peptide epitopes during virulent *Salmonella* infection. The latter observation supports the use of T cell peptide vaccination as a potential strategy to combat ongoing virulent *Salmonella* infection. Our finding that the frequency of immunodominant *Salmonella*-specific CD4^+^ T cell responses can be used as an immunological predictor for the disease activity opens new avenues for diagnosis of *Salmonella* infection and may aid in the rational design of vaccines.

An important role for CD4^+^ T cell immunity is also found in other persistent infections that are bacterial (e.g. *Mycobacterium tuberculosis*), parasitic (e.g. *Leishmania major*) or viral (e.g. cytomegalovirus) [47], [48], [49], [50]. Thus far, little is known about CD4^+^ T cell responses generated in response to virulent *Salmonella*. Here we identified novel CD4^+^ T cell epitopes to facilitate the study of *Salmonella*-specific T cell immunity. More than 1400 epitopes were predicted, of which, three elicited significantly elevated responses in *Salmonella*-infected C57BL/6 mice. To our knowledge, we provide here for the first time the identification of epitopes that are able to monitor CD4^+^ T cell responses during virulent *Salmonella* infection directly *ex vivo*. These epitopes were found in different *Salmonella* enzymes. Since these epitopes are located in conserved enzymes that are also present in other microbes, it might be that in these microbes the same epitopes elicit specific CD4^+^ T cell responses. Based on high *in vivo* expression levels Rollenhagen et al. identified *Salmonella* antigens for protective immunity but the epitope specificity remained unknown [51]. Other studies have identified epitopes in FliC (15) and recently two epitopes in the type III secretion system were reported [21]. FliC is transcriptionally switched off once *Salmonella* is inside macrophages, which renders this response not suitable to track T cell responses during virulent infection [52], [53]. Yet, immunization with FliC protein results in development of protective CD4^+^ T cell immunity [19], [54]. The epitopes within the type III secretion system proteins were discovered using a non-virulent *Salmonella* model and the CD4^+^ T cell responses to these epitopes in this model displayed similar kinetics as compared to the EutC_243–257_-specific response during virulent *Salmonella* infection [21].

One reason for the gap in knowledge regarding development of T cell immunity to *Salmonella* is that traditional CD4^+^ T cell antigen screening and epitope mapping methods are hampered by the large genome size of bacteria including *Salmonella.* Bioinformatic predictions of peptide:MHC class II binding have been successfully used to identify peptide epitopes genome wide in viruses such as vaccinia virus and mouse cytomegalovirus 31,55, and also in the known B cell antigen targets of the intracellular bacterium *Coxiella burnetti* [56]. The rate of identified CD4^+^ T cell epitopes in the tested *Salmonella* peptides was only ∼0.2% (3/1443), which clearly is a low efficiency, and in fact lower than the identified CD4^+^ T cell epitopes in vaccinia virus (∼0.7% hit rate) and much lower than the identification in mouse cytomegalovirus and the focused search in *Coxiella burnetti* (both ∼7% hit rate). It is likely that additional T cell epitopes recognized in virulent *Salmonella* exist, as our screen was directed at identifying the most dominant peptide responses, which was necessitated by the relatively high level of background responses in the animals with virulent infection. Also, the limitation to screen only the top 0.5% of peptides will necessarily exclude some responses. Finally, the algorithms used were based on a limited training dataset, and if repeated nowadays would have picked a very different and presumably better set of candidate peptides, as evidenced by the fact that the FLiC peptide was not included in our screen, but would now be included. In summary, we believe that the responses identified are some of the most dominant, but we believe that multiple additional T cell epitopes exist. Another possible limitation to our screen is that it selected only IFN-γ producing T cells. Additional CD4^+^ T cell epitopes may exist that do not induce IFN-γ and/or TNF, but do trigger production of other cytokines.

The role of CD4^+^ T cells during *Salmonella* infection is likely two-fold: 1) direct effector cell activity and 2) providing help for B cells and CD8^+^ T cells. Since IFN-γ is a critical cytokine for controlling *Salmonella* infection, it is likely that CD4^+^ T cell derived IFN-γ plays a particularly important role [12]. Besides producing IFN-γ, direct killing activity of CD4^+^ T cells, which has been described in lymphocytic choriomeningitis virus [57], West Nile virus [58] and dengue virus [59] infection, might also be a function of these cells. Since MHC class II-expressing macrophages are an important reservoir of *Salmonella* [60], [61], it might be that these cells are the targets. Further studies are required to discriminate between these different effector functions.

In conclusion, we demonstrate the feasibility of MHC class II binding predictions to identify CD4^+^ T cell responses during virulent *Salmonella* infection. The prospect of predicting the burden of *Salmonella* infections based on the responses against these newly identified CD4^+^ T cell epitopes could lead to new diagnostic tools and reagents for tracking and analyzing *Salmonella*-specific T cell responses during *Salmonella* disease or after vaccination. While the exact epitopes identified in mice are not likely to be recognized in humans due to differences in MHC specificity, their source proteins are more likely to be prevalently recognized in humans [62]. Further studies that aim to predict human CD4^+^ T cell epitopes (e.g. HLA DR1/3/4) could translate into direct clinical applications. The results obtained provide novel insights into the mechanisms of T cell immunity to virulent *Salmonella* and could therefore aid in the diagnostics of *Salmonella* infection and in a rational development of (subunit) vaccines to *Salmonella* serovars.

## Materials and Methods

### Ethics Statement

Animal experiments performed at the La Jolla Institute for Allergy and Immunology (LIAI) were approved by the LIAI Animal Care and Use Committee (number: AF10-057-022807-A2) and performed according the recommendations in the Guide for the Care and Use of Laboratory Animals of the National Institutes of Health and the guidelines of the Association for Assessment and Accreditation of Laboratory Animal Care International (AAALAC). Animal experiments performed at Leiden University Academic Center (LUMC) were approved by the Animal Experiments Committee of the LUMC (number: 10225) and performed according to the guide to animal experimentation set by the LUMC and to the Dutch Experiments on Animals Act that serves the implementation of ‘Guidelines on the protection of experimental animals’ by the Council of Europe.

### Mice

C57BL/6 mice were purchased either from The Jackson Laboratory (Bar Harbor, ME) and maintained at the Department of Laboratory Animal Care at LIAI or were purchased from Charles River (France) and maintained at the Central Animal Facility of LUMC. All mice were housed in specific pathogen-free conditions and used at 8–12 weeks of age.

### *Salmonella* infection and quantification of disease activity

The virulent *Salmonella enterica* serovar Typhimurium (*S*. Typhimurium) strain LT2 was obtained from the American Type Culture Collection (ATCC). For infections, *S*. Typhimurium was grown to mid-log phase in Luria-Bertani media at 37°C with shaking and subcultures were setup to grow to early-log phase (OD 600 nm of ∼0.25). The bacterial concentration was estimated using a spectrophotometer and the bacteria were diluted in PBS. Mice were orally infected with 1×10^7^ *Salmonella* colony forming units (CFU) in 200 µl PBS using a gavage needle. Eight hours before infection, all mice were fasted and 5 minutes prior to infection 100 µl sodium bicarbonate was administered by oral gavage to neutralize stomach acid. In all infection experiments, the actual bacterial dose was confirmed by plating serial dilutions onto freshly prepared MacConkey agar plates. To determine bacterial colonization *in vivo*, spleens from infected mice (day 7, 14, 21 and 28 post-infection) were homogenized in 0.5× PBS and the number of recoverable *Salmonella* CFU enumerated by plating serial dilutions onto freshly prepared MacConkey agar plates. Alanine aminotransferase (ALT) levels in serum samples from naive and *Salmonella*-infected mice were analyzed at the department of Clinical Chemistry (LUMC) according to standard methodology.

### CD4^+^ T cell epitope prediction

The entire *Salmonella* proteomes of *S*. Typhimurium strain LT2 [28] and *S*. Typhi strain CT18 [29] were scanned for conserved 15-mer peptide sequences that are predicted, using a consensus approach as described [31], to have a high-affinity binding capacity for the MHC class II molecule H2 I-A^b^. Briefly, predictions were obtained from the ARB and SMM-align tool on the immune epitope database (IEDB) website [63], and all peptides were ranked according to their predicted affinity by each method. To construct a consensus from the prediction methods, the median of the ranks was used to select peptides for screening. A set of 1443 different peptides with the highest median ranks corresponding to the top ∼0.5% scoring peptides were selected for screening and were synthesized as crude material (A&A Labs, San Diego, CA) and used in initial screening experiments. Peptides AhpC_154–168_, EutC_243–257_, STM1540_262–276_ (see Table 1 for amino acid sequences), and FliC_429–443_ (VQNRFNSAITNLGNT [19]) used in subsequent experiments were re-synthesized as purified material.

### Peptide vaccination

For peptide vaccination, all mice were challenged with 1×10^7^ CFU of virulent *S*. Typhimurium (strain LT2) provided orally as described above. At day 5 post-infection, mice were vaccinated with a mixture of the following peptides: AhpC_154–168_, EutC_243–257_ and STM1540_262–276_. The mixed peptide vaccines contained either 8 µg (∼5 nmol) or 40 µg (∼25 nmol) of each of the peptides and were injected subcutaneously in the flank in a total volume of 200 µl PBS. Control mice were injected with PBS alone. For prophylactic vaccination, mice received 14 days prior *Salmonella* infection the same mixture of peptides (i.e.:AhpC_154–168_, EutC_243–257_, STM1540_262–276_; 40 µg each peptide) supplemented with 10 µg CpG (ODN 1826, InvivoGen). Bacterial colonization was determined as described above.

### In vivo antibody treatment

Hybridomas were cultured in Life Technologies Protein-Free Hybridoma Medium-II (Invitrogen, San Diego, CA), and monoclonal antibodies were purified by dialysis of supernatants. To deplete CD4^+^ T cells, 100 µg of anti-mouse CD4 mAb (clone GK1.5) was injected intraperitoneally (i.p.) on days 0, 3, 6 and 9 post-infection. To deplete CD8^+^ T cells, 100 µg anti-mouse CD8 mAb (clone 2.43) was injected i.p. on days 0, 3, 6 and 9 after infection. All antibodies were administered in 200 µl PBS.

### Preparation of peptide-loaded dendritic cells

Bone marrow cells were harvested from wild-type C57BL/6 mice and resuspended in culture medium (Iscove's Modified Dulbecco's Medium (IMDM), 10% heat inactivated fetal calf's serum, 2 mM L-Glutamine, and 2-mercaptoethanol), which was supplemented with 200 ng/ml Flt-3 ligand (Peprotech). After 10 days, dendritic cells were harvested and either loaded with peptides (10 µg/ml) for 90 minutes at 37°C or not loaded with peptides (medium control). After extensive washing, peptide-loaded and unloaded dendritic cells were used for ELISPOT assays and intracellular cytokine staining.

### ELISPOT Assay

Antigen-specific IFN-γ recall responses were measured by enzyme-linked immunospot (ELISPOT). 2×10^5^ CD4^+^ T cells, purified by CD4 magnetic microbeads (Miltenyibiotec), were cultured with 4×10^4^ peptide-loaded dendritic cells in a total volume of 100 µl culture medium in Multiscreen 96-well plates (Millipore) that were precoated overnight at 4°C with 5 µg/ml rat-anti-mouse IFN-γ. Background values were determined by culturing purified CD4^+^ T cells with unloaded dendritic cells. After 16 h incubation at 37°C, the plates were washed with PBS containing 0.05% Tween 20, followed by incubation with 1 µg/ml biotinylated IFN-γ antibody at room temperature for two hours. After washing, extravidine (Sigma) and BCIP/NBT were used to visualize spots. The frequency of IFN-γ producing cells was quantified by ELISPOT Readers (Autoimmun Diagnostika GmbH, Strassberg, Germany). Wells were considered positive if they yielded values >2 times above mean background plus two standard deviations.

### Flow cytometric analysis and intracellular staining

Spleens were harvested and single-cell suspensions were prepared by mincing through 70 µm cell strainers (BD Biosciences). Erythrocytes were lysed in a hypotonic (0.82%) ammonium chloride buffer. For cell surface staining, cells were resuspended in staining buffer (PBS +1% FCS +0.05% sodium azide) and incubated with fluorescent conjugated antibodies for 30 minutes at 4C° in presence of Fc block (monoclonal antibody to mouse CD16/CD32). To determine the activation status on conventional CD4^+^ T cells, regulatory T cells were excluded by staining for intracellular Foxp3 using the anti-mouse Foxp3 staining kit (eBioscience). For determination of intracellular cytokine expression, 4×10^5^ purified CD4^+^ T cells were cultured with 8×10^4^ peptide-loaded dendritic cells in 96-well flat-bottom plates in a total volume 200 µl culture medium for 8 h of which the last 6 h were in presence of 1 µg/ml brefeldin A. Background was determined by culturing purified CD4^+^ T cells with unloaded dendritic cells (medium control). After incubation, cells were transferred to U-bottom 96-well plates, and the cell surface was stained with fluorescent conjugated antibodies at 4C° for 0.5 h in staining buffer. After washing, cells were fixed with Fix/perm buffer for 1 h followed by intracellular cytokine staining at 4C° for 1 h in Perm/Wash buffer (BD Biosciences). Cells were washed and resuspended in staining buffer before analysis. Flow cytometric acquisition was performed with a BD LSR II flow cytometer and data was analyzed using FlowJo software (Tree Star). Fluorochrome-conjugated monoclonal antibodies specific for CD3, CD4, CD8, CD62L, CD69, KLRG1, IFN-γ, TNF, IL-2, IL-10 and IL-17 were purchased from BD Biosciences (San Diego, CA) or eBioscience (San Diego, CA).

### Statistical analysis

Statistical significance between groups was determined with the Mann-Whitney test and correlations were analyzed with linear regression. We used Prism GraphPad Software for all statistical analyses.

## Supporting Information

Figure S1 **Optimization of cytokine expression in** ***Salmonella*** **-specific CD4^+^ T cells.** C57BL/6 mice were infected orally with 1×10^7^ virulent *Salmonella* Typhimurium and at day 14 post-infection the IFN-γ and TNF expression in *Salmonella*-specific CD4^+^ T cells was determined by intracellular staining after restimulation with peptide-loaded dendritic cells (DCs). (A) Titration of peptide dose. CD3^+^CD4^+^ T cells were stimulated with DCs (DC:T cell ratio  = 1∶5) that were loaded with different concentrations of STM1540_262–276_ peptide or not loaded with peptide (medium). Graph shows the percentage of IFN-γ^+^TNF^+^ cells within the CD3^+^CD4^+^ T cell population plotted against the indicated peptide concentrations. (B) Titration of DC:T cell ratio. 4×10^5^ CD3^+^CD4^+^ T cells were stimulated with different amounts of DCs that were loaded with 10 µg/ml STM1540_262–276_ peptide or not loaded with peptide (medium). Graph shows the percentage of IFN-γ^+^TNF^+^ cells within the CD3^+^CD4^+^ T cell population *versus* the different DC:T cell ratios. Shown are representative analyses of two independent experiments with three mice per experiment.
(EPS)
